# Sex Chromosomes Regulate Nighttime Sleep Propensity during Recovery from Sleep Loss in Mice

**DOI:** 10.1371/journal.pone.0062205

**Published:** 2013-05-01

**Authors:** J. Christopher Ehlen, September Hesse, Lennisha Pinckney, Ketema N. Paul

**Affiliations:** Neuroscience Institute, Morehouse School of Medicine, Atlanta, Georgia, United States of America; Kent State University, United States of America

## Abstract

Sex differences in spontaneous sleep amount are largely dependent on reproductive hormones; however, in mice some sex differences in sleep amount during the active phase are preserved after gonadectomy and may be driven by non-hormonal factors. In this study, we sought to determine whether or not these sex differences are driven by sex chromosome complement. Mice from the four core genotype (FCG) mouse model, whose sex chromosome complement (XY, XX) is independent of phenotype (male or female), were implanted with electroencephalographic (EEG) and electromyographic (EMG) electrodes for the recording of sleep-wake states and underwent a 24-hr baseline recording followed by six hours of forced wakefulness. During baseline conditions in mice whose gonads remained intact, males had more total sleep and non-rapid eye movement sleep than females during the active phase. Gonadectomized FCG mice exhibited no sex differences in rest-phase sleep amount; however, during the mid-active-phase (nighttime), XX males had more spontaneous non-rapid eye movement (NREM) sleep than XX females. The XY mice did not exhibit sex differences in sleep amount. Following forced wakefulness there was a change in the factors regulating sleep. XY females slept more during their mid-active phase siestas than XX females and had higher NREM slow wave activity, a measure of sleep propensity. These findings suggest that the process that regulates sleep propensity is sex-linked, and that sleep amount and sleep propensity are regulated differently in males and females following sleep loss.

## Introduction

Reproductive hormones, particularly in females, exert regulatory influences on the sleep-wake cycle. As early as 1969, Colvin et al. [1] reported that estrogen treatment in ovariectomized (OVX) mice reduces nighttime rapid eye movement (REM) sleep amount. Two years later, Branchey et al., [2] reported that combined injections of estrogen and progesterone reduce daily NREM and REM sleep amounts in OVX rats. Since then, studies in rats and mice have repeatedly reported that OVX increases sleep amount while exogenous estrogen administration reduces it [3], [4], [5], [6], [7]. In the majority of these studies, the influences of OVX and estrogen on sleep occurred mostly during the active phase (i.e. the dark phase of a 24-hr LD cycle in nocturnal species).

In 2006, we reported that male mice exhibited more daily NREM sleep than female mice and that gonadectomy (GDX) in males and females eliminated this sex difference [8]. OVX in females was responsible for the majority of the reduction of the sex difference, with castration (CAST) in the males contributing to a lesser degree. However, some of the sex differences reported in that study were not altered by GDX. Most notably, during the mid-active phase GDX males exhibited more NREM sleep than GDX females. This sex difference occurred near the peak of active-phase sleep amount and sleep propensity in rodents, commonly known as siesta sleep in humans and fruit flies 9,10.

Sleep propensity is an inclination to sleep that is driven by: 1) the circadian timing system and 2) homeostatic pressure to sleep that builds during extended wakefulness. Although sleep amount is an indicator of sleep propensity, the most reliable measure is NREM slow wave activity (SWA), which measures the spectral power of delta (.5–4 Hz) oscillations in electroencephalographic (EEG) recordings during NREM sleep. In 2006, we reported that GDX reduced sex differences in absolute values of NREM SWA, but that the dissipation of SWA during recovery from sleep deprivation occurred more quickly in GDX males than in GDX females.

In the current study, we sought to examine the role of sex linkage in regulating sleep phenotypes by testing the hypothesis that sex differences that remain in GDX mice are driven by sex chromosome complement. To accomplish our goal we took advantage of the four core genotype (FCG) mouse line in which the sex chromosome complement is independent of gonadal sex [11], [12]. This mouse line has helped reveal the influences of sex linkage on a number of behavioral and neuroanatomical phenotypes including the sexual differentiation of brain cells [13], agonistic and asocial behaviors [14] and habitual responses to alcohol reinforcement [15]. We implanted EEG/electromyographic (EMG) electrodes in adult gonadally-intact and GDX FCG mice and measured sleep-wake architecture and SWA under baseline conditions and after 6 hrs of forced wakefulness, paying specific attention to sex differences that occurred during the active phase. We report that sex differences in sleep propensity during recovery from sleep loss are largely driven by sex chromosome complement.

## Materials and Methods

### Animals

This study was carried out in strict accordance with the recommendations in the Guide for the Care and Use of Laboratory Animals of the National Institutes of Health. All protocols and procedures were approved by the Morehouse School of Medicine Institutional Animal Care and Use Committee (NIH OLAW Assurance Number A3944-01). All efforts were made to minimize pain and suffering. Adult FCG breeder mice on a C57BL/6J background were kindly donated by Dr. Arthur Arnold (UCLA, Los Angeles, CA) and the line was bred at Morehouse School of Medicine and maintained on a 12-hour light:12-hour dark (12L:12D) schedule throughout the study. This mouse line was initiated by a spontaneous deletion of the testis determining gene “*Sry*” from the Y chromosome of MF-1 outbred mice and the subsequent insertion of the *Sry* transgene onto an autosome [16]. Without this gene testis development does not occur, and mice lacking *Sry* develop ovaries. When XX mice have an *Sry* autosomal transgene, (these mice are designated XXM) they develop normal functioning testes. Conversely, XYF mice lack the *Sry* autosomal transgene and consequently develop ovaries. The FCG mouse line is comprised of four primary genotypes: XXF (XX mice lacking *Sry*, with ovaries), XYF (XY mice lacking *Sry*, with ovaries, XXM (XX mice with *Sry* and testes) and XYM (XY mice with *Sry* and testes). This study examined gonadally intact (XYM (n = 3), XYF (n = 3), XXM (n = 3) and XXF (n = 3), and GDX (XYM (n = 7), XYF (n = 8), XXM (n = 10) and XXF (n = 10)) mice. Food and water were available ad libitum.

### Gonadectomy(GDX)

Animals (8–10 weeks of age) were deeply anesthetized with an intraperitoneal injection of ketaset (80 mg/kg) and xylazine (8 mg/kg). In male mice, a vertical incision was made at the midline of the abdomen, and fatty and connective tissue were removed to expose the inner sacs that encase the testicles. A second incision was made through each casing, and the testes fat pad and epididymes were expressed to the outside and removed. The area was cleaned, and the incisions were closed using silk suture. In female mice, an incision was made dorsally through the skin and muscle just above the location of the ovary. The ovary was expressed to the surface and ligated using a silk suture. The ovary was removed, the uterine horn was returned to the cavity, and the muscle and skin layers were sutured.

### Recording-Implant Surgery

Immediately following GDX, EEG and EMG electrodes for polysomnographic recording of sleep–wake states were implanted in anesthetized mice. A prefabricated head mount (Pinnacle Technologies, KS) was used to position four stainless steel epidural screw electrodes. The first 2 electrodes (frontal and ground) were located 1.5 mm anterior to Bregma and 1.5 mm on either side of the central suture whereas the second 2 electrodes (parietal/occipital and common reference) were located 3.5 mm posterior to bregma and 1.5 mm on either side of the central suture. Electrical continuity between the screw electrodes and headmount was aided by the use of silver epoxy. EMG activity was monitored using stainless-steel Teflon coated wires integrated into the headmount and inserted bilaterally into the nuchal muscle. The headmount (integrated 2x3 pin grid array) was secured to the skull with dental acrylic. In order to control for pain, over the next 36 h mice were given Buprenex (buprenorphine; 2 mg/kg subcutaneously) once every 12 h. All procedures were performed on a heating pad and mice were allowed to recover for at least 14 days before being transferred to sleep recording chambers. A separate cohort of FCG mice with intact gonads was also implanted with recording electrodes.

### EEG/EMG recordings

Following recovery (at least 14 days), the mice were placed in a sleep-recording chamber and connected to a lightweight tether connected to a low-resistance commutator mounted over the cage (Pinnacle Technologies, KS). This enabled complete freedom of movement throughout the cage. Except for the recording tether, conditions in the recording chamber were identical to those in the home cage. Mice were allowed a minimum of 7 additional days to acclimate to the tether. Recording of EEG and EMG waveforms began at zeitgeber time (ZT) 0 (light onset in 12:12 LD). Data acquisition was performed on a PC running Sirenia Acquisition software (Pinnacle Technologies, KS), a software system designed specifically for polysomnographic recording in rodents. EEG signals were low-pass filtered with a 40 Hz cutoff and collected continuously at a sampling rate of 400Hz. After collection, all waveforms were classified in two-second epochs by a trained observer (using both EEG leads and EMG) as wake (low-voltage, high-frequency EEG; high-amplitude EMG), NREM sleep (high-voltage, mixed-frequency EEG; low- amplitude EMG) or rapid-eye movement (REM) sleep (low-voltage EEG with a predominance of theta activity [6–10 Hz]; very low amplitude EMG). EEG epochs determined to have artifact (interference caused by scratching, movement, eating, or drinking) were excluded from analysis. Artifact comprised less than five percent of all recordings used for analysis. Analysis of slow wave activity (SWA) was accomplished by applying a Fast Fourier Transformation to raw EEG waveforms. Only epochs classified as NREM sleep were included in this analysis. SWA was measured as spectral power in the 0.5 to 4 Hz frequency range.

### Forced wakefulness

Following a 24-hr baseline recording, mice were sleep deprived during the first 6 hours of the light phase (ZT 0–6) by gentle handling (introduction of novel objects into the cage, tapping on the cage and when necessary delicate touching) and allowed an18-hr recovery opportunity (ZT 6–0). Four of the animals from the baseline recording were excluded from recovery analysis because of deterioration of the EEG or EMG signal (1 XXM, 1 XYF, and 2 XXFs).

### Statistics

2×2 comparisons (males vs. females; XY mice vs. XX mice) were conducted in gonadally-intact animals and were analyzed using two-way ANOVAs with phase (light or dark) as the within-subjects variable. Post-hoc analyses of these 2×2 comparisons were conducted using paired t-tests with a Bonferroni correction (corrected α_c_ = .025), since there were only two groups to compare. To compare across the FCG genotypes, repeated measures ANOVAs were used to detect between- (genotype; XYM, XYF, XXM, and XXF) and within- (time; averaged in 2-hr intervals) factor differences. Since these analyses included more than two groups they required multiple comparisons, therefore post hoc analysis was conducted using Tukey tests to follow-up variance of main effects and interactions. All t-statistics are reported as the absolute value. 2×2 comparisons (Table 1) were made for genetic sex (XYM & XYF vs. XXM & XXF) and for gonadal sex (XYM & XXM vs. XXM & XXF). Significance in all tests was defined as p≤0.05.

**Table 1:** 2×2 comparisons of FCG mice with intact gonads.

		XY	XX	Male	Female
**TS**	L	905.1±29.3	901.9±15.8	900.1±20.2	907.3±24.2
	D	444.5±60.4	470.9±52.4	523.9±43.7	378.3±35.2*
**NREMS**	L	798.3±26.4	780.7±12.0	785.8±19.3	792.0±19.2
	D	420.5±386.2	436.9±47.8	490.6±40.7	353.45±33.0*
**REMS**	L	113.2±7.4	124.7±12.3	123.0±11.0	115.3±10.9
	D	24.0±2.0	34.0±5.2	33.3±5.4	24.8±2.3

XY mice (XYM; XYF) and XX mice (XXM; XXF) and gonadal males (XYM; XXM) and females (XYF; XXF). Sex differences in TOTAL SLEEP and NREM sleep amount are similar to what was previously reported in intact wild-type mice (Paul et al., 2006). Data are presented mean ± sem.

## Results

### FCG mice with intact gonads exhibit sex differences in sleep amount

First we investigated sex differences in mice with intact gonads. For this analysis, 2×2 comparisons of sex and chromosome complement were sufficient. During 24-hr recording of baseline sleep-wake states, males (XYM & XXM) had 69.2 min. more total sleep (F_1,10_ = 11.1; p = 0.008), and 65.5 min. more NREM sleep (F_1,10_ = 5.7; p = 0.038) than females (XYF & XXF), but no differences in REM sleep amount (F_1,10_ = 1.3; p = 0.29; Table 1). Sex by phase (light or dark) interactions for total sleep (F_1,10_ = 12.8; p = 0.005) and NREM sleep (F_1,10_ = 9.5; p = .012) revealed that these sex differences were predominant during the active (dark) phase. Follow-up paired t-tests revealed the sex differences during the dark phase (total sleep: t_10_ = 3.9; p = 0.003; NREM sleep: t_10_ = 3.0; p = 0.013) but not the light phase (total sleep: t_10_ = 0.3; p = 0.77; NREM sleep: t_10_ = .46; p = 0.65). These effects of gonadal sex are similar to those previously reported in wild-type mice [8].

We then investigated the effect of genetic sex (sex chromosome complement) in intact FCG mice by comparing XY mice (XYM & XYF) to XX mice (XXM &XXF) during 24-hr baseline sleep recording. There were no significant effects of sex chromosome complement on total sleep, NREM sleep, or REM sleep during baseline recording (Table 1). These results reveal that genetic sex does not actively influence sex differences in spontaneous sleep amounts in mice with intact gonads.

### GDX FCG mice exhibit sex differences in sleep amount during the active phase

**Figure 1:**
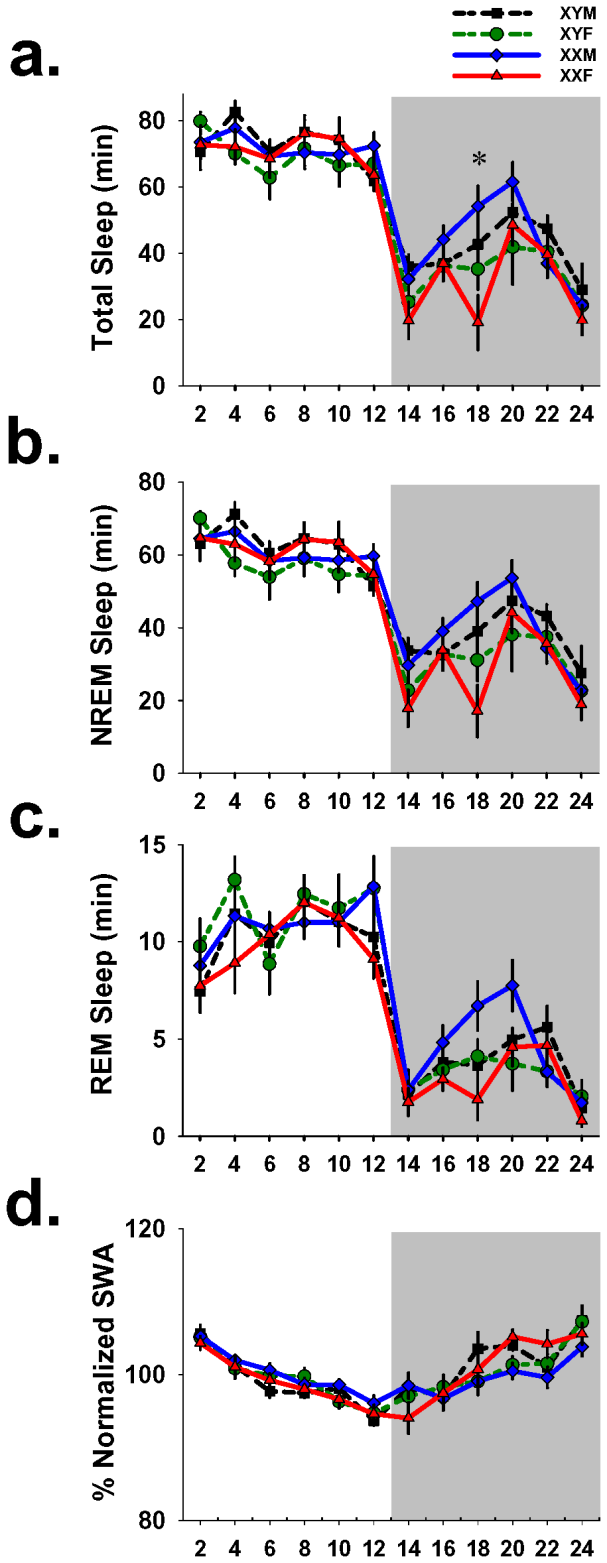
Genotype had an effect on total sleep during the active phase. 24-hr baseline sleep records of GDX FCG mice averaged into 2-hr intervals. Graphs show minutes spent in: (a) total sleep, (b) NREM sleep and (c) REM sleep. Figure (d) shows normalized NREM SWA for 2-hr intervals as percentages of 24-hr SWA. During the dark phase at ZT 16–18 XXM mice consistently exhibited more sleep than XXF mice (indicated by asterisk). Error bars represent mean ± sem. *p<0.05 Tukey posthoc test (XXM≠XXF). Shaded area represents the 12-hr dark phase.

Next we investigated the effect of sex chromosome complement on sleep in GDX FCG mice. During 24-hr baseline recording, repeated measures ANOVA revealed main effects of genotype on total sleep (F_3,31_ = 3.2; p = .04), but not on NREM sleep (F_3,31_ = 2.3; p = .09), or REM sleep (F_3,31_ = 2.4; p = .09). Post hoc analyses of total sleep averaged into 2-hr intervals revealed no differences between any of the four genotypes during the 12-hr light phase. Post-hoc analyses revealed that during a single 2-hr interval between ZT 16–18 just prior to the peak in active-phase sleep amount, GDX XXMs had 39.3 min. more total sleep (p = .01, Tukey) than GDX XXFs (Figure 1a). Interestingly, this sex difference was only significant in the XX mice and not the XY mice (p = .06, Tukey). A similar sex difference at the same interval was previously reported in GDX wild-type mice [8]. Baseline NREM SWA was normalized by dividing the SWA of each 2-hr interval by the total SWA for 24 hrs. A repeated measures ANOVA revealed no effects of genotype on SWA.

### Active phase sleep amount after forced wakefulness is partially dependent on sex chromosome complement

**Figure 2:**
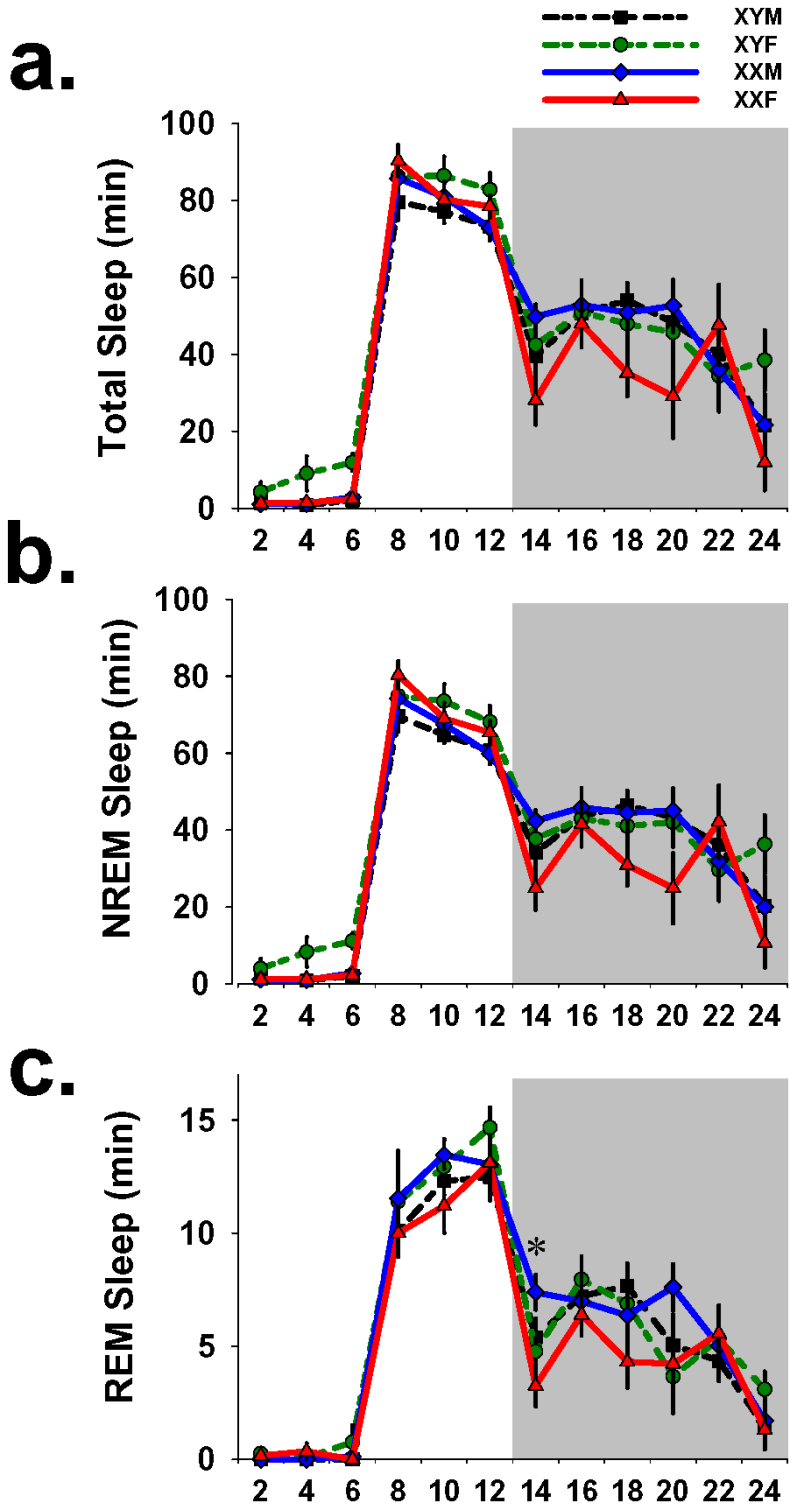
Absolute recovery sleep amount was not sensitive to genotype after sleep loss. 24-hr sleep records of GDX FCG mice during 6 hrs of forced wakefulness and 18 hrs of recovery sleep averaged into 2-hr intervals. Graphs show minutes spent in: (a) total sleep, (b) NREM sleep and (c) REM sleep. During the dark phase at ZT 12–14 XXM mice exhibited more REM sleep than XXF mice (indicated by asterisk). Error bars represent mean ± sem. * p<0.05 Tukey posthoc test (XXM≠XXF). Shaded area represents the 12-hr dark phase.

During 18 hrs of recovery sleep after forced wakefulness there were no main effects of genotype on absolute amounts of total sleep (F_3,26_ = 2.5; p = .08) or NREM sleep (F_3,26_ = 1.5; p = .22), but there was a main effect of genotype on REM sleep (F_3,26_ = 5.9; p = .003). Posthoc analysis of REM sleep revealed that at ZT 12–14 XXMs had 4.1 min. more REM sleep than XXFs (p = .004, Tukey; Figure 2c). Since all animals averaged a total of 5.2±.5 minutes of REM sleep during this interval, a difference of 4 minutes between genotypes is substantial.

**Figure 3:**
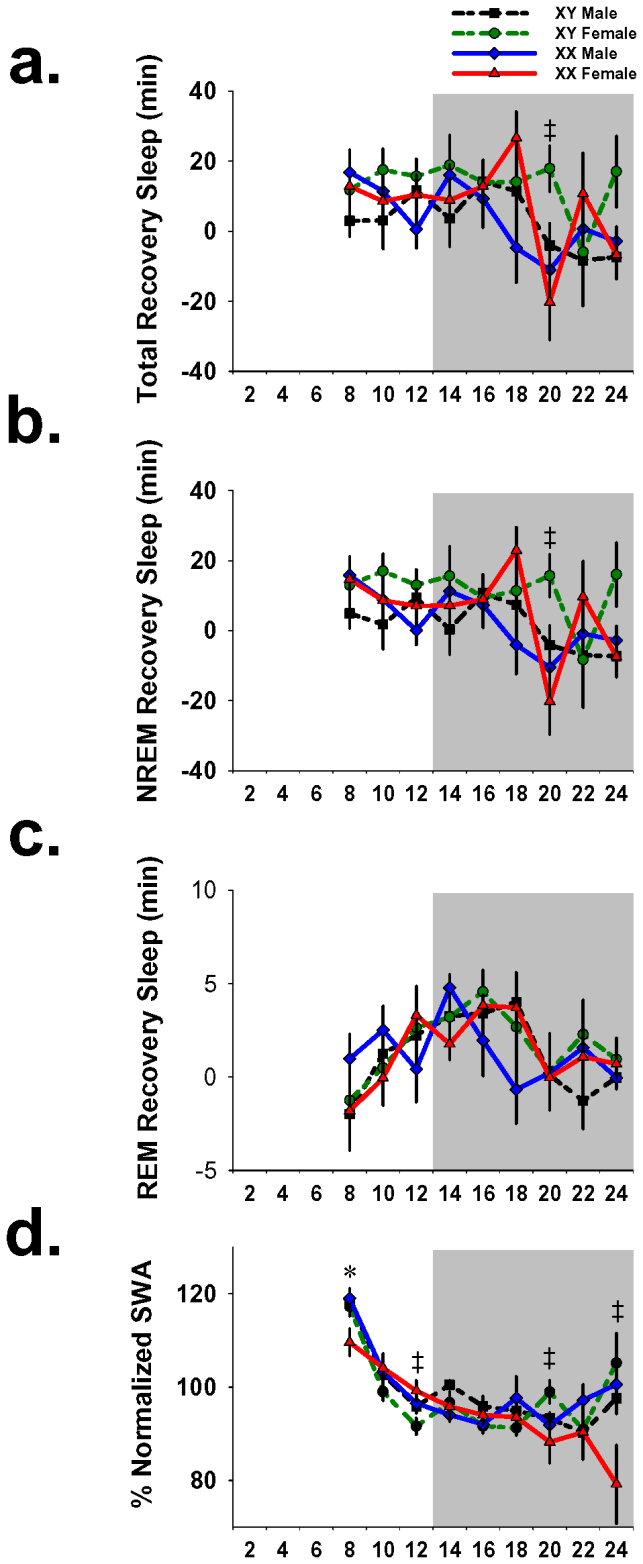
Genotype had effects on recovery sleep amount and slow wave activity normalized to baseline. 18 hrs of recovery sleep amount averaged into 2-hr intervals and then normalized to baseline by expressing the difference in minutes. Graphs show percentage of recovery sleep for (a) total sleep, (b) NREM sleep and (c) REM sleep. Figure (d) shows normalized NREM SWA for 2-hr intervals as percentages of 24-hr SWA. Error bars represent mean ± sem. *p<0.05 Tukey posthoc test (XXM≠XXF). ‡p<0.05 Tukey posthoc test (XYF≠XXF). Shaded area represents the 12-hr dark phase.

To obtain a more accurate measure of the homeostatic response to sleep loss we expressed sleep recovery as the difference in minutes from corresponding time-interval of baseline sleep in the same animal. This is a measure of sleep gained (or lost) during recovery from forced wakefulness. There were no main effects of genotype on total recovery sleep (F_3,26_ = 2.9; p = .05), NREM recovery sleep (F_3,26_ = 2.7; p = .05) or REM recovery sleep (F_3,26_ = .5; p = .66); however, there were genotype x time interactions for total sleep (F_3,26_ = 4.5; p = .01) and NREM sleep (F_3,26_ = 1.6; p = .04). Post hoc analysis revealed that during 2 hrs at the peak of active phase siesta sleep, XYFs had 38.2 min. more total recovery sleep (p = .043, Tukey) and 35.9 min. more NREM recovery sleep (p = .027, Tukey) than XXFs (Figure 3). Interestingly, at the same time interval (ZT 18–20) there was little variability in REM sleep amount between groups (F_3,26_ = .007; p = .999; Figure 3c). This finding suggests that the influences of genetic sex during this time interval extend almost exclusively to NREM sleep regulation.

NREM SWA during recovery was normalized by dividing the SWA of each 2-hr interval by the corresponding total baseline SWA recorded in the same animal. Repeated measures ANOVA revealed a main effect of genotype on recovery NREM SWA (F_3,26_ = 4.9; p = .01) and a genotype x time interaction (F_3,26_ = 2.0; p = .007). Posthoc analysis revealed effects at four time intervals (Figure 3d). Immediately following forced wakefulness, during ZT 6–8, XXFs had the lowest SWA of the groups and had 9.3% less SWA than XXMs (p = .036, Tukey). Conversely, four hours later during ZT 10–12, the same XXFs had the highest SWA and had 7.6% more SWA than XYFs (p = .017, Tukey). The first 6 hrs of recovery sleep represent the most rapid dissipation of NREM SWA after sleep deprivation. The observation that XXFs started this span with the lowest SWA and ended with the highest suggests that the rate of SWA dissipation is slower in this genotype than in the other three. In fact, this was the only genotype that exhibited a steady SWA decline during the entire 18-hr recover period and finished the recovery (ZT 22–24) with the lowest SWA (p = .046, Tukey; Figure 3d). The other time interval that exhibited an effect of genotype was at ZT 18–20, during which mice exhibit the most siesta sleep. During this interval, XYFs had 10.7% more SWA than XXFs. This observation suggests that during siesta sleep, females bearing the Y chromosome have a higher sleep propensity than females bearing the X chromosome. The same effect was not present in males.

## Discussion

The results of this study provide evidence that sex-linked genes, or factors linked to the sex chromosomes, actively influence the ability to recover from sleep loss. It is important to emphasize that this effect of sex chromosomes appears to be dependent on sleep loss and is particularly apparent during the mid-active phase (ZT 18–20), during which GDX XY females had almost 40 minutes more recovery sleep than GDX XX females. This time interval and the adjacent intervals are of particular interest because it is when mice exhibit the most active-phase sleep and elevated active-phase SWA. The mid-late active phase is also when humans are most likely to experience elevated propensity for siesta sleep [9]. This was not the only sex chromosome-influenced phenotype that was revealed by sleep loss. Spectral analysis of recovery NREM sleep uncovered four different time points during which NREM SWA in XY females was different from XY males. XX females began the recovery period with the lowest NREM SWA of all groups; however, six hours later they had the highest SWA, suggesting their sleep propensity accumulated more slowly during sleep loss, and dissipated more slowly during the initial stages of recovery sleep. Then during siesta sleep, XY females had higher SWA than XX females. This is particularly interesting since XY females also had more recovery sleep during this time interval. This enhancement in sleep propensity in XY females during the active phase, reflected by increases in sleep amount and NREM SWA, suggests two things: 1) sex chromosome complement influences the ability to recover from sleep loss, and 2) the effect of sex chromosomes on sleep propensity following sleep loss are most apparent during mid-active phase siesta sleep. This finding is important because it suggests that after sleep loss, siesta sleep is genetically influenced, and it implicates the X or Y chromosome as the potential origin of this genetic link. This finding is also important in the context of reports that siesta napping during the active phase may be an important tool for properly recovering, and reducing the impairments that result, from sleep loss [17], [18].

FCG mice with intact gonads in the current study exhibited sex differences similar to previous reports in wild-types [8]. Specifically, during the active phase gonadal males (regardless of sex chromosome complement) exhibited more total sleep and NREM sleep than gonadal females at the expense of wakefulness during baseline recording. After GDX, FCG mice continued to exhibit sex differences in sleep and wake amount during the mid-active phase (ZT 16–18), a finding that was also previously reported in wild-types. When we examined the effect of genotype on mid-active phase sleep we discovered that this sex difference was only significant in XX mice, not in XY mice. Similar effects were present for both NREM and REM sleep. In addition, during the mid-late active phase (ZT 18–20), when mice typically obtain most of their siesta sleep, XX females had higher SWA than XX males.

The observation that GDX males continue to sleep more than GDX females during the mid-active phase of baseline recording, but only in the XX mice, results from two potential possibilities. The first is that expressing the *Sry* gene on an XX background is sufficient to increase mid-active phase sleep amount. The second is that there may be lingering effects of gonadal hormones, or some effects of sex phenotype, that only express themselves in XX mice. Such effects are commonly associated with organizational effects of reproductive hormones. The observation that this sex difference is not observed after sleep deprivation suggests that under conditions of high homeostatic sleep pressure, direct effects of sex chromosomes on sleep propensity mask the phenotypic sex differences observed during baseline sleep.

Sex chromosome complement had a number of revealing influences on NREM SWA during recovery from sleep loss. NREM SWA measures homeostatic sleep pressure, which builds with increasing duration of wakefulness and dissipates during subsequent NREM sleep. When mice are sleep deprived during the first six hours of the light phase, the most rapid dissipation of SWA typically occurs during the remaining six hours of the light phase. During this span, SWA in XX females declined more slowly than any other genotypes. This finding in similar to our previous report which indicates that after sleep loss SWA declines more slowly in GDX females than in males. This suggests that the presence of the Y chromosome, or a male phenotype, enhances SWA dissipation after sleep loss. In fact, while SWA began increasing during the late dark phase in the other genotypes, it continued to fall in the XX females, who ended the recovery period with lower SWA than the other genotypes. This suggests that the homeostatic ability to dissipate sleep pressure after sleep loss is regulated differently in mice that lack the male genotype or phenotype.

Over the past several years a number of studies have reported that women are more likely than men to suffer negative health outcomes from insufficient daily sleep. [19], [20], [21], [22], [23]. Underlying reports of higher morbidity in sleep-deprived women is the hypothesis that sex differences in the homeostatic sleep regulation causes the accumulation of sleep propensity over time to become more debilitating in women [24]. Women are more likely to report sleep disturbances and greater levels of perceived sleepiness, and are less likely to report feeling rested in the morning than men. They also report fewer actual episodes of behavioral sleepiness during the active phase (i.e., falling asleep against one's will and/or at inappropriate times) compared to men. Moreover, several studies report that men have a higher predisposition for afternoon napping than women [25], [17]. The results from the current study suggest that sex-linked genes have direct regulatory influence s on the sleep-wake cycle and are at least partially responsible for increased compulsions to nap during the active phase (daytime in humans, nighttime in mice) when sleep pressure is high. These findings implicate the mid-late active phase as a potential target to relieve sleep pressure in chronically sleep-deprived women.

## References

[1] Colvin GB, Whitmoyer DI, Sawyer CH (1969) Circadian sleep-wakefulness patterns in rats after ovariectomy and treatment with estrogen. Exp Neurol 25: 616–625. 5391423 10.1016/0014-4886(69)90104-6

[2] Branchey M, Branchey L, Nadler RD (1971) Effects of estrogen and progesterone on sleep patterns of female rats. Physiol Behav 6: 743–746. 4337177 10.1016/0031-9384(71)90267-8

[3] Yamoaka S (1980) Modification of circadian sleep rhythms by gonadal steroids and the neural mechanisms involved. Brain Res 185: 385–398. 6101987 10.1016/0006-8993(80)91076-8

[4] Li H, Satinoff E (1996) Changes in circadian rhythms of body temperature and sleep in old rats. Am J Physiol 269: R208–214. 10.1152/ajpregu.1995.269.1.R208 7631895

[5] Fang J, Fishbein W (1996) Sex differences in paradoxical sleep: influences of estrus cycle and ovariectomy. Brain Res 734: 275–285. 8896835

[6] Pawlyk AC, Alfinito PD, Johnston GH, Deecher DC (2008) Subchronic 17alpha-ethinyl estradiol differentially affects subtypes of sleep and wakefulness in ovariectomized rats. Horm Behav 53: 217–224. 17976600 10.1016/j.yhbeh.2007.09.018

[7] Schwartz MD, Mong JA (2011) Estradiol suppresses recovery of REM sleep following sleep deprivation in ovariectomized female rats. Physiol Behav 104: 962–971. 21722658 10.1016/j.physbeh.2011.06.016 PMC3183102

[8] Paul KN, Dugovic C, Turek FW, Laposky AD (2006) Diurnal sex differences in the sleep-wake cycle of mice are dependent on gonadal function. Sleep 29: 1211–1223. 17040009 10.1093/sleep/29.9.1211

[9] Monk TH, Buysse DJ, Carrier J, Billy BD, Rose LR (2001) Effects of afternoon "siesta" naps on sleep, alertness, performance, and circadian rhythms in the elderly. Sleep. 24: 680–687. 10.1093/sleep/24.6.680 11560181

[10] Wijnen H, Young MW (2008) The right period for a Siesta. Neuron 60: 943–946. 19109901 10.1016/j.neuron.2008.12.009

[11] De Vries GJ, Rissman EF, Simerly RB, Yang LY, Scordalakes EM, et al (2002) A model system for study of sex chromosome effects on sexually dimorphic neural and behavioral traits. J Neurosci 22: 9005–9014. 12388607 10.1523/JNEUROSCI.22-20-09005.2002 PMC6757680

[12] Arnold AP, Chen X (2009) What does the "four core genotypes" mouse model tell us about sex differences in the brain and other tissues? Front Neuroendocrinol 30: 1–9. 19028515 10.1016/j.yfrne.2008.11.001 PMC3282561

[13] Carruth LL, Reisert I, Arnold AP (2002) Sex chromosome genes directly affect brain sexual differentiation. Nat Neurosci 5: 933–934. 12244322 10.1038/nn922

[14] McPhie-Lalmansingh AA, Tejada LD, Weaver JL, Rissman EF (2008) Sex chromosome complement affects social interactions in mice. Horm Behav 54: 565–570. 18590732 10.1016/j.yhbeh.2008.05.016 PMC2561329

[15] Barker JM, Torregrossa MM, Arnold AP, Taylor JR (2010) Dissociation of genetic and hormonal influences in sex differences in alcoholism-related behaviors. J Neurosci 30: 9140–9144. 20610747 10.1523/JNEUROSCI.0548-10.2010 PMC2921163

[16] Mahadevaiah SK, Odorisio T, Elliott DJ, Rattigan A, Szot M, et al (1998) Mouse homologues of the human AZF candidate gene RBM are expressed in spermatogonia and spermatids, and map to a Y chromosome deletion interval associated with a high incidence of sperm abnormalities. Hum Mol Genet 7: 715–727. 9499427 10.1093/hmg/7.4.715

[17] Naska A, Oikonomou E, Trichopoulou A, Psaltopoulou T, Trichopoulos D (2007) Siesta in healthy adults and coronary mortality in the general population. Arch Intern Med 167: 296–301. 17296887 10.1001/archinte.167.3.296

[18] Zaregarizi M, Edwards B, George K, Harrison Y, Jones H, et al (2007) Acute changes in cardiovascular function during the onset period of daytime sleep: comparison to lying awake and standing. J Appl Physiol 103: 1332–1338. 17641220 10.1152/japplphysiol.00474.2007

[19] Cappuccio FP, Stranges S, Kandala N-B, Miller MA, Taggart FM, et al (2007) Gender-specific associations of short sleep duration with prevalent and incident hypertension. The Whitehall II study. Hypertension 50: 693–700. 17785629 10.1161/HYPERTENSIONAHA.107.095471 PMC3221967

[20] Miller MA, Kandala NB, Kivimaki M, Kumari M, Brunner EJ, et al (2009) Gender differences in the cross-sectional relationships between sleep duration and markers of inflammation: Whitehall II study. Sleep 32: 857–864. 19639748 PMC2706900

[21] Gangwisch JE, Malaspina D, Babiss LA, Opler MG, Posner K, et al (2010) Short sleep duration as a risk factor for hypercholesterolemia: analyses of the National Longitudinal Study of Adolescent Health. Sleep 33: 956–961. 20614855 10.1093/sleep/33.7.956 PMC2894437

[22] Kronholm E, Laatikainen T, Peltonen M, Sippola R, Partonen T (2011) Self-reported sleep duration, all-cause mortality, cardiovascular mortality and morbidity in Finland. Sleep Med 12: 215–221. 21317033 10.1016/j.sleep.2010.07.021

[23] Lyytikäinen P, Rahkonen O, Lahelma E, Lallukka T (2011) Association of sleep duration with weight and weight gain: a prospective follow-up study. J Sleep Res 20: 298–302. 21199039 10.1111/j.1365-2869.2010.00903.x

[24] Armitage R (2007) Sleep and circadian rhythms in mood disorders. Acta Psychiatr Scand Suppl 433104–115. 10.1111/j.1600-0447.2007.00968.x 17280576

[25] Reimão R, Souza JC, Gaudioso CE, Guerra HD, Alves AD, et al (2000) Siestas among Brazilian Native Terena adults: a study of daytime napping. Arq Neuropsiquiatr 58: 39–44. 10770864 10.1590/s0004-282x2000000100006

